# Nonoperative Treatment of Intra-articular Calcaneus Fractures Is Associated With Lower Rates of Certain Long-term Adverse Events Compared to Operative Management: A Retrospective Database Analysis

**DOI:** 10.1177/24730114261450622

**Published:** 2026-05-31

**Authors:** Ava M. McKane, N. Reid Kiritsis, Karl V. Mohr, Omkar S. Anaspure, Conor N. O’Neill, Albert T. Anastasio

**Affiliations:** 1 Wake Forest University School of Medicine, Winston-Salem, NC, USA; 2 Perelman School of Medicine at the University of Pennsylvania, Philadelphia, PA, USA; 3 Duke University Medical Center, Durham, NC, USA; 4 Atrium Health Wake Forest Baptist Medical Center, Medical Center Boulevard, Winston-Salem, NC, USA

**Keywords:** calcaneus fracture, Nonoperative treatment, intra-articular calcaneus

## Abstract

**Background:**

Management of displaced intra-articular calcaneus fractures remains controversial. While operative techniques are well-described, few large-scale studies have compared long-term outcomes between operative vs nonoperative treatment. Conservative management may limit soft-tissue complications, while operative treatment may improve alignment and potentially reduce the risk of posttraumatic arthritis.

**Methods:**

The TriNetX Research Network, including 140 million patient records, was used to identify patients with displaced intra-articular calcaneus fractures using *ICD-10* codes S92.061, S92.062, S92.063 over a 20-year period. Patients were stratified into operative (*CPT*: 28415, 28420, 28406) and nonoperative (*CPT*: 28400, 28405) cohorts. Patients with polytrauma or incomplete records were excluded. Propensity score matching adjusted for demographics and comorbidities, yielding 1260 patients per group. Primary outcomes included any adverse event within 90 days and posttraumatic osteoarthritis, nonunion, malunion, and conversion to subtalar fusion at 2 and 5 years. Statistical analyses included *t* test, χ^2^, and odds ratios with 95% CIs (*P* < .05).

**Results:**

Baseline demographics and comorbidities were balanced between groups. At 90 days, operative treatment was associated with significantly higher rates of overall adverse events (8.5% vs 3.6%, OR 2.51, *P* < .001) and lower emergency department utilization (11.5% vs 21.6%, OR 0.47, *P* < .001). At 5 years, operative patients experienced higher rates of posttraumatic osteoarthritis (6.1% vs 3.8%, OR 1.64, *P* = .008) and implant-related complications (3.6% vs 1.3%, OR 2.71, *P* < .001). Nonunion, malunion, and subtalar fusion rates were similar at all time points.

**Conclusion:**

Operative treatment was associated with higher rates of certain complications. Findings should be interpreted cautiously given limitations, including coding variations, lack of surgical specifics, and inability to stratify by fracture severity. The observed associations do not establish causality, as fracture severity is a critical determinant of outcomes and treatment selection, although the findings are important for informing future studies. Prospective studies incorporating fracture classification, cost-analysis, and patient-reported outcomes are needed.

**Level of Evidence:**

Level III, retrospective analysis.

## Introduction

Intra-articular calcaneus fractures are high-energy injuries, most often caused by motor vehicle accidents or falls from height in otherwise healthy adults, accounting for 1% to 2% of all fractures.^1-3^ They occur predominantly in men and individuals who work in fields involving manual labor.^4,5^ Calcaneus fractures pose significant impacts on patient quality of life and can cause delays in the ability to work, leading to significant economic losses.^6^ As a result of economic pressure, healing time can be complicated, as patients may be forced to use the injured foot for work before complete healing can be achieved. These injuries can severely impact quality of life, particularly because of the high risk of posttraumatic arthritis following the injury and the weightbearing nature of the calcaneus and subtalar joint, with some studies showing postfracture functional impairment exceeding that of myocardial infarction across most categories of the 36-item Short Form Health Survey (SF-36) scoring.^7^

Current literature has shown some differences in surgical vs nonsurgical treatment of intra-articular fractures, such as variability in recovery time; however, many of these studies have yet to be performed using large patient cohorts.^6,8,9^ One study looking exclusively at male patients found that operative treatment had better outcomes than nonoperative when assessed on the American Orthopaedic Foot & Ankle Society ankle hindfoot scale, Modified Rowes Scale, and visual analog scale.^10^ In contrast, another study showed that operative treatment led to almost double the time to return to work, and associated costs to the patient.^8^ To complicate matters, factors such as workers compensation status and gender have been shown to be significant modulators of outcomes when comparing surgical to conservative intervention, with some studies showing that a lack of workers compensation and female gender have been associated with better outcomes in the surgical cohorts.^6^ Although randomized trials exist, they are underpowered, and recent reviews still call for large-scale, longitudinal comparisons, which is what this study aims to provide.^11-13^ Additionally, propensity score matching in a real-world data set adds unique value by mimicking trial balance while still leveraging a large population.

Because of the substantial economic, social, and occupational consequences of intra-articular calcaneus fractures and associated time needed for recovery, finding differences in optimal treatment options is essential to clinical decision making. To address the lack of large-scale evidence in the use of conservative vs operative treatment of intra-articular calcaneal fractures, we used the TriNetX database to evaluate outcomes of surgical vs nonoperative management in a large cohort with multiyear follow-up. We hypothesized that nonoperative management would have similar or better outcomes than surgical management of intra-articular calcaneal fractures.

## Methods

### Data Collection and Patient Selection

This study used the TriNetX database, a research platform that allows access to deidentified health care records from health care organizations across the globe. The query was run on the Research network, which contains information from 105 health care organizations and >140 million patients. The search was performed on May 23, 2025, and we collected records dating back to 20 years. The data set solely contains deidentified health care information without individual or identifiable patient details, so the present study was exempt from institutional review board approval.

Two cohorts were constructed for comparing treatment for displaced intra-articular calcaneus fractures. The operative cohort included patients with a diagnosis of displaced intra-articular fracture of the calcaneus (*ICD-10*: S92.061, S92.062, S92.063) who underwent open or percutaneous fixation, identified using *CPT* codes 28415, 28420, and 28406. To better ensure that these fractures were isolated lower limb fractures and enhance comparison of the cohorts, patients were excluded if they were polytraumatized, such as those with associated fractures of the tibia, fibula, talus, or other tarsal/metatarsal bones within 14 days. The nonoperative cohort included patients with the same *ICD-10* diagnoses of an intra-articular calcaneus fracture who underwent closed treatment (*CPT* codes 28400 or 28405) and had no record of percutaneous or open fixation or concomitant fractures in adjacent bones. Patients whose procedures occurred more than 20 years before the search date or had incomplete records were excluded. Patients with missing data or values required for inclusion were automatically excluded by TriNetX platform, and no additional data handling changes were made. Propensity score matching was performed on age, sex, race, body mass index (BMI), type 2 diabetes mellitus, nicotine dependence, chronic obstructive pulmonary disease (COPD), chronic kidney disease (CKD), heart failure, and chronic ischemic heart disease. Matching resulted in 1260 patients in each cohort. After propensity score matching, there was still a residual difference in BMI (*P* = .015, standardized mean difference [SMD] 0.122). To evaluate the influence of the residual BMI imbalance after matching (SMD = 0.122, *P* = .015), E-values were calculated for all statistically significant outcomes. The E-value represents the minimum strength of association an unmeasured confounder would require with both the treatment assignment and outcome to nullify an observed association.^14^

### Outcome Measures

Postoperative outcomes were assessed at 90 days, 2 years, and 5 years following the index surgery using predefined time windows within the TriNetX database. These intervals reflect database-based follow-up capture rather than structured, clinical protocol follow-up. Outcomes were identified using *ICD-10* and *CPT* codes. The primary outcomes were the rate of any adverse event (AAE) (T81.3, I26, I21, I63, I82.40, L76.22, T81.4, A41.9, J18; *ICD-10-PCS*: 30233N1) and medical complications within 90 days, and the rates of posttraumatic osteoarthritis (M19.17) and delayed healing, malunion, or nonunion at 2- and 5-year follow-up, identified via *ICD-10* codes S92.001 to S92.009 and S92.061 to S92.063 with 7th-character modifiers G (delayed healing), K (nonunion), or P (malunion); and S99.001 to S99.009 and S99.091 to S99.092 with modifiers K or P. Additional outcomes examined included surgical site infection, wound dehiscence, venous thromboembolism (deep vein thrombosis and/or pulmonary embolism), myocardial infarction, cerebral infarction, sepsis, blood transfusion, hematoma, emergency department visits, and inpatient admissions.

### Statistical Analysis

The baseline demographics and comorbidities of patients in each cohort were assessed using *t* tests and χ^2^ tests. Statistical analyses of baseline demographics and postoperative outcomes were conducted within TriNetX. The outcomes are presented as rates and odds ratios (ORs) with *P* values and 95% CIs. Statistical significance was assessed at *P* = .05. It is important to note that several Table 3 cells show exactly “10” with identical ORs which is consistent with TriNetX’s count suppression for values ≤10. Specifically, this refers to rates of transfusions, acute myocardial infarction, cerebral infarct, pulmonary embolism, deep vein thrombosis, venous thromboembolism, prosthesis mechanical complication, and conversion to subtalar fusion. Statistical comparison of these values is unreliable, so we did not report the associated *P* values or odds ratios.

## Results

This was an analysis of 5226 patients who underwent treatment for displaced intra-articular calcaneus fractures, with matched cohorts of 1260 patients each. Before matching, the mean age was 46.4 ± 14.7 years in the operative cohort and 48.8 ± 16.1 years in the conservative cohort. After propensity score matching, all baseline characteristics were well balanced, with only BMI remaining statistically different between groups (*P* = .015; SMD = 0.122), and all SMDs were less than 0.2, indicating acceptable covariate balance. Full baseline characteristics are detailed in Table 1. A total of 1364 patients were available for complete follow-up at 2 years for an attrition rate of 45.9%, and 689 were available at 5 years for an attrition rate of 72.7% (Table 2).

**Table 1.** Baseline Characteristics Before and After Propensity Score Matching for Operative vs Conservative Treatment of Displaced Intra-articular Calcaneus Fractures.

	Before Propensity Score Matching			After Propensity Score Matching			
	Open/Percutaneous Fixation (n = 3956)	Closed Treatment (n = 1270)	*P* Value	Open/Percutaneous Fixation (n = 1260)	Closed Treatment (n = 1260)	*P* Value	SMD
Age at index, y, mean ± SD	46.4 ± 14.7	48.8 ± 16.1	<.001^a^	47.9 ± 14.9	48.7 ± 16.0	.185	0.053
BMI, mean ± SD	26.6 ± 5.4	26.0 ± 5.4	.008^a^	26.7 ± 5.4	26.1 ± 5.4	.015^a^	0.122
Male, n (%)	2817 (71.2)	870 (68.7)	.015^a^	876 (69.5)	868 (68.9)	.730	0.014
White, n (%)	2862 (72.3)	899 (71.0)	.091	896 (71.1)	897 (71.2)	.965	0.002
Black or African American, n (%)	352 (9.0)	143 (11.3)	.018^a^	150 (11.9)	139 (11.0)	.492	0.027
Asian, n (%)	115 (2.9)	32 (2.5)	.431	35 (2.8)	32 (2.5)	.710	0.015
Type 2 diabetes mellitus, n (%)	266 (6.8)	122 (9.6)	.001^a^	109 (8.7)	116 (9.2)	.625	0.019
Nicotine dependence, n (%)	1,237 (31.3)	372 (29.4)	.115	333 (26.4)	369 (29.3)	.110	0.064
Chronic ischemic heart disease ()	206 (5.3)	79 (6.2)	.197	60 (4.8)	77 (6.1)	.135	0.060
Heart failure, n (%)	63 (1.6)	37 (2.9)	.003^a^	27 (2.1)	31 (2.5)	.595	0.021
Chronic kidney disease, n (%)	102 (2.6)	45 (3.6)	.082	38 (3.0)	42 (3.3)	.649	0.018
COPD, n (%)	154 (3.9)	77 (6.1)	.001^a^	61 (4.8)	73 (5.8)	.287	0.042

Abbreviations: BMI, body mass index; COPD, chronic obstructive pulmonary disease.

a Statistically significant, *P* < .05.

**Table 2.** Patient Follow-up and Attrition Rates at 2 and 5 Years in Operative and Nonoperative Cohorts.

	Operative	Nonoperative	Combined
Total (matched)	1260	1260	2520
≥2-y follow-up	644 (51.1)	720 (57.1)	1364 (54.1)
Lost to follow-up at 2 y	616 (48.9)	540 (42.9)	1156 (45.9)
≥5-y follow-up	308 (24.4)	381 (30.2)	689 (27.3)
Lost to follow-up at 5 y	952 (75.6)	879 (69.8)	1831 (72.7)

**Figure 1.**
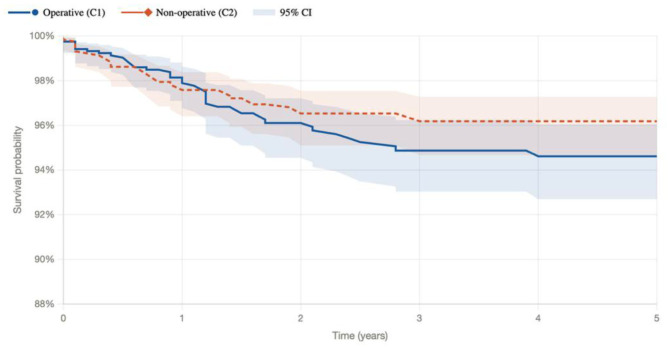
Kaplan-Meier curve demonstrating time-to-event outcomes for conversion to subtalar fusion in operative and nonoperative (conservative) treatment groups.

The overall rate of any adverse event at 90 days was higher in the operative cohort compared to the conservative cohort: 8.5% vs 3.6% (*P* < .001), respectively. At 90 days, the operative group had significantly higher odds of wound dehiscence (4.8%) and surgical site infection (3.3%) (*P* < .001). In contrast, the conservative cohort experienced higher rates of emergency department (ED) visits (21.6% vs 11.5%; OR 0.47, *P* < .001). There were no significant differences between groups in rates of conversion to subtalar fusion (Figure 1), venous thromboembolism, deep vein thrombosis, pulmonary embolism, or sepsis at 90 days (Table 3).

**Table 3.** Ninety-Day Complication Rates, *P* Values, and Odds Ratios for Operative vs Conservative Treatment of Displaced Intra-articular Calcaneus Fractures.^a^

	Operative Cohort, n (%)	Conservative Cohort, n (%)	*P* Value	Odds Ratio (95% CI)
Any adverse event^b^	107 (8.5)	45 (3.6)	<.001^c^	2.506^c^ (1.753, 3.582)
Dehiscence	60 (4.8)	12 (1.0)	<.001^c^	5.200^c^ (2.784, 9.713)
Transfusion	≤10 (0.8)	≤10 (0.8)	—	—
Acute myocardial infarction	≤10 (0.8)	≤10 (0.8)	—	—
Cerebral infarct	≤10 (0.8)	≤10 (0.8)	—	—
Pulmonary embolism	≤10 (0.8)	≤10 (0.8)	—	—
Deep vein thrombosis	≤10 (0.8)	≤10 (0.8)	—	—
Venous thromboembolism	12 (1.0)	≤10 (0.8)	.668	1.202 (0.517, 2.792)
Surgical site infection	41 (3.3)	≤10 (0.8)	<.001^c^	4.204^c^ (2.097, 8.431)
Emergency department visit	145 (11.5)	272 (21.6)	<.001^c^	0.472^c^ (0.379, 0.588)
Hematoma	≤10 (0.8)	0 (0.0)	—	—
Sepsis	≤10 (0.8)	11 (0.9)	—	—
Inpatient admission	107 (8.5)	96 (7.6)	.421	1.125 (0.844, 1.500)
Infected implant	23 (1.8)	13 (1.0)	.093	1.784 (0.899, 3.537)
Implant mechanical complication	≤10 (0.8)	≤10 (0.8)	—	—
Posttraumatic osteoarthritis	12 (1.0)	≤10 (0.8)	—	—
Conversion to subtalar fusion	≤10 (0.8)	≤10 (0.8)	—	—

a Cells with a dash denote unreliable statistical tests due to count suppression for instances ≤10 in TriNetX. The surgical site infection (SSI) odds ratio and *P* value should be interpreted with caution, as the conservative cohort count is suppressed.

b Any adverse event: dehiscence, deep vein thrombosis, pulmonary embolism, myocardial infarction, cerebral infarct, transfusion, hematoma, SSI, sepsis.

c Denotes statistical significance, *P* < .05.

After 2 years, the rates of implant infection and mechanical implant complications were 4.0% and 2.7% in the operative group, whereas rates of nonunion/malunion, posttraumatic osteoarthritis, and conversion to subtalar fusion were not statistically significant between groups (Table 4).

**Table 4.** Two-Year Complication Rates, *P* Values, and Odds Ratios for Operative vs Conservative Treatment of Displaced Intra-articular Calcaneus Fractures.

	Operative Cohort, n (%)	Conservative Cohort, n (%)	*P* Value	Odds Ratio (95% CI)
Infected implant	51 (4.0)	14 (1.1)	<.001^a^	3.754^a^ (2.067, 6.818)
Implant mechanical complication	34 (2.7)	10 (0.8)	<.001^a^	3.467^a^ (1.705, 7.047)
Delayed healing/ nonunion/ malunion	79 (6.3)	74 (5.9)	.677	1.072 (0.773, 1.487)
Posttraumatic osteoarthritis	59 (4.7)	41 (3.3)	.066	1.461 (0.973, 2.193)
Conversion to subtalar fusion	30 (2.4)	32 (2.5)	.850	0.953 (0.579, 1.569)

a Denotes statistical significance, *P* < .05.

By 5 years, 4.1% of patients in the operative group experienced a prosthesis infection, and 3.6% saw mechanical complications from their hardware. Posttraumatic osteoarthritis was significantly more common in the operative cohort (6.1% vs 3.8%, OR 1.64, *P* = .008) (Table 5). However, it is noteworthy that rates of conversion to subtalar fusion did not reach statistical significance between groups across all time points (Table 5).

**Table 5.** Five-Year Complication Rates, *P* Values, and Odds Ratios for Operative vs Conservative Treatment of Displaced Intra-articular Calcaneus Fractures.

	Operative Cohort, n (%)	Conservative Cohort, n (%)	*P* Value	Odds Ratio (95% CI)
Infected implant	52 (4.1)	18 (1.4)	<.001^a^	2.970^a^ (1.728, 5.106)
Implant mechanical complication	45 (3.6)	17 (1.3)	<.001^a^	2.708^a^ (1.541, 4.758)
Delayed healing/nonunion/malunion	84 (6.7)	79 (6.3)	.686	1.068 (0.777, 1.467)
Posttraumatic osteoarthritis	77 (6.1)	48 (3.8)	.008^a^	1.643^a^ (1.136, 2.378)
Conversion to subtalar fusion	35 (2.8)	36 (2.9)	.904	0.971 (0.606, 1.557)

a Denotes statistical significance, *P* < .05.

A sensitivity analysis using E-values demonstrated that the observed significant associations were robust to the BMI imbalance present after matching. E-values for statistically significant outcomes ranged from 2.67 to 9.87, with E-values for the 95% CI bounds ranging from 1.53 to 5.01 (Table 6). Given that the mean BMI difference between matched groups was 0.6 (26.7 vs 26.1), the residual BMI imbalance is unlikely to account for the magnitude of association required to undermine the observed findings.

**Table 6.** E-Value Sensitivity Analysis for Statistically Significant Outcomes Addressing Residual BMI Imbalance (SMD = 0.122) Following Propensity Score Matching.

	Timepoint	OR (95% CI)	E-Value (Point Estimate)^a^	E-Value (95% CI Bound)^b^
Any adverse event	90-d	2.51 (1.75, 3.58)	4.45	2.90
Dehiscence	90-d	5.20 (2.78, 9.71)	9.87	5.01
Surgical site infection	90-d	4.20 (2.10, 8.43)	7.87	3.61
Emergency department visit	90-d	0.47 (0.38, 0.59)	3.66	2.79
Infected implant	2-y	3.75 (2.07, 6.82)	6.97	3.55
Implant mechanical complication	2-y	3.47 (1.71, 7.05)	6.39	2.80
Infected implant	5-y	2.97 (1.73, 5.11)	5.39	2.85
Mechanical complication	5-y	2.71 (1.54, 4.76)	4.86	2.45
Posttraumatic osteoarthritis	5-y	1.64 (1.14, 2.38)	2.67	1.53

Abbreviations: BMI, body mass index; OR, odds ratio; SMD, standardized mean difference.

a E-value = OR + √(OR × (OR − 1)) for OR ≥ 1; 1/OR substituted prior to calculation for OR < 1.

b The CI bound column reflects the 95% CI limit closest to the null. All calculations used 1/OR prior to applying the formula for outcomes with OR < 1.

## Discussion

In our cohort of 2520 matched patients, nonoperative treatment of displaced intra-articular calcaneus fractures was associated with lower rates of adverse events at both short- and long-term follow-up compared with operative treatment. As anticipated, the operative cohort experienced significantly higher rates of wound complications in 90 days, including surgical site infections and dehiscence.^15^ Conservative treatment was also associated with lower long-term rates of posttraumatic arthritis. Calcaneus fractures are debilitating injuries associated with chronic pain, functional limitations, and reduced quality of life.^16,17^ Surgical intervention has traditionally been the standard of care for displaced or intra-articular calcaneus fractures, with the goal of restoring joint congruity and proper alignment.^18^ Various techniques have been described, including percutaneous cannulated screw fixation and open reduction internal fixation.^19-21^ However, few large-scale investigations have evaluated the outcomes of conservative management.

Our findings differ from SooHoo et al,^22^ which reported lower wound complication rates; potential explanations include differences in coding era, case mix, and comorbidity profile. We observed higher rates of 90-day adverse events in the operative cohort. Whereas their study reported wound infection and thromboembolic event (deep vein thrombosis / pulmonary embolism) rates of 1.03% and 0.25%, respectively, our analysis found higher rates of surgical site infection (3.3%) and thromboembolic events (0.8%). Several factors may account for these differences. The TriNetX database aggregates data from a wide range of hospital settings and geographic regions, potentially including a greater proportion of patients with comorbidities such as diabetes, obesity, and smoking, all of which are known risk factors for postoperative complications. In contrast, the SooHoo study cohort was derived from a single state and may reflect a more uniform population. Additionally, their data set spans from 1995 to 2005, which pre-dates modern perioperative protocols and coding standards, further explaining observed differences.

Most notably, operative treatment was associated with higher rates of posttraumatic osteoarthritis, male union, and non-union at 5 years postoperatively. These findings align with prior studies reporting substantial rates of posttraumatic arthritis following fixation.^23^ When evaluating rates of conversion to subtalar fusion, no significant difference was observed between operative and conservative groups, further suggesting the long-term benefits of fixation may be limited. Although smaller-scale studies have suggested that operative treatment lowers the risk of late subtalar arthrodesis, our large-scale findings challenge this view.^24^ Limited statistical power in smaller cohorts may impair the ability to detect true equivalence. Differences in outcome definitions may also contribute, as prior studies often report arthrodesis as a secondary outcome with relatively limited follow-up. Additionally, selection bias may be amplified in smaller samples, particularly when surgical fixation is preferentially offered to patients with more severe injuries. Together, these results highlight the need for individualized treatment selection and suggest that conservative management may be associated with fewer complications in select patient populations—particularly those with elevated surgical risk or poor soft tissue. Prior literature has reported similar long-term outcomes between treatment groups, such as little to no difference in posttraumatic osteoarthritis; however, this was in the setting of a younger, predominantly male cohort, which may not accurately reflect the broader patient demographics seen with calcaneal fractures.^12^ Cost considerations cannot be overlooked as well, as literature has shown the financial burden of operative care in the first year posttreatment to be greater than those that were treated nonoperatively. Brauer et al^9^ found a Can$2150 difference between groups when considering treatment costs and initial follow-up within the first year. These discrepancies underscore the importance of individualized treatment selection based on patient comorbidities and overall health status.

There is limited literature directly comparing malunion and nonunion rates between treatment strategies, highlighting the value of large, matched data sets in addressing this gap. Comorbidities such as high BMI, diabetes mellitus, and smoking status, are important considerations when determining appropriate treatment strategies.^25^ As a further consideration, the TriNetX research network does not allow for granular evaluation of calcaneus-fracture specific complication rates, such as the rate of varus malunion, dorsiflexion impingement from loss of calcaneal pitch, complications related to lateral wall blow-out, or persistent peroneal tendon instability from superior peroneal retinaculum injury. Operative management may still provide certain advantages over nonoperative treatment with regard to these fracture-specific complications. Outside of these considerations, our findings suggest that nonoperative management may confer certain benefits in recovery and posttraumatic arthritis rates; however, consistent with prior studies, treatment should remain individualized. Surgical intervention remains a viable option and may be appropriate depending on patient-specific characteristics and clinical presentation.

Additionally, we observed lower emergency department utilization in the operative cohort. This may reflect structured postoperative follow-up and improved pain management, reducing unscheduled visits. Differences in health care access and utilization captured by TriNetX may have also contributed to this trend.

The TriNetX research network, which spans 20 years and more than 100 health care organizations, provided a large data set, which enhanced the generalizability of our findings.^26^ However, limitations should be acknowledged. TriNetX does not capture patient-reported outcome measures, and reliance on diagnosis-based outcomes does not allow for assessment of radiographic healing, complication severity, or treatment success. *ICD-10* codes cover the presence of a diagnosis but cannot fully distinguish major from minor complications or quantify functional recovery. Reliance on administrative codes such as *ICD-10* and *CPT* introduces susceptibility to coding inaccuracies, incomplete documentation, or misclassification.^27^ Small but nonzero rates of wound complications observed in the nonoperative cohort likely reflect coding error, misclassification of treatment status, or wound issues unrelated to surgical intervention, such as traumatic soft tissue injuries. Although propensity matching mitigates confounding, residual bias may persist because of unmeasured variables, such as insurance status, functional outcomes, or surgeon expertise. It is worth noting that 68% of the operative cohort was excluded during propensity score matching. Although this reduced confounding, it limits generalizability. Findings may not extend to all surgical patients, especially those with multiple comorbidities or complex presentations. This trade-off between balance and representativeness is inherent to propensity score matching.

Deidentified data preclude chart-level validation to assess clinical variables such as degree of displacement, making it impossible to differentiate fracture severity across Sanders class II, III, and IV, introducing potential treatment selection bias. The Sanders classification is strongly prognostic, with type III fractures carrying a significantly higher risk of subsequent subtalar fusion.^28^ Treatment decisions are also influenced by factors not captured by coding data, such as Sanders classification, soft tissue condition, surgeon preference, and patient-specific demands, where more severe injuries are more likely managed operatively, whereas nonoperative care favors minimally displaced fractures with a more favorable baseline prognosis. Fracture energy, which correlates to Sanders classification, cannot be discerned through administrative coding and represents a major source of confounding in our analysis. Although propensity matching accounts for demographics, it does not capture fracture-specific characteristics, so observed outcomes may reflect injury severity instead of true effect of management. Without granular fracture data, definitive conclusions cannot be drawn; however, our findings provide insight into intra-articular calcaneal fractures as a broader category. Observed differences in complication rates may reflect underlying fracture severity rather than a causal effect of treatment modality. The odds of developing posttraumatic arthritis is largely determined by the severity and energy of the initial fracture, with more severe injuries conferring greater risk regardless of treatment approach.^29^

Infected implant outcomes were identified using *ICD-10* codes T84.5, T84.6, and T84.7 within the 2- and 5-year follow-up windows in the TriNetX database. However, these codes are not anatomically specific to calcaneus implants and may capture orthopaedic implant infections at any site. For example, a patient with a history of calcaneus fracture who subsequently develops a knee implant infection would be included. Additionally, the proportion of patients with available long-term data at 2- and 5-year follow-up is not reported. Database attrition from patients leaving participating networks may introduce an additional selection bias if those patients that are retained are systematically different from those lost to follow-up.

Given that deep infection beyond 90 days is relatively uncommon, the persistent nonzero infection rates observed at later time points may reflect coding limitations and represent a potential source of bias. Certain outcomes, such as wound complications and postoperative arthritis, are inherently procedure-specific and may be influenced by differences in follow-up intensity. Further, grouping open reduction internal fixation (*CPT* 28415, 28420) and percutaneous fixation (*CPT* 28406) into a single operative cohort should be acknowledged. The inability to perform subgroup analysis by surgical approach confounds our analysis, as the observed 4.8% dehiscence rate reflects differing risk profiles across a heterogenous mix of techniques. Nonetheless, these outcomes remain relevant when evaluating the full spectrum of clinical complications associated with each management strategy.

Another key limitation of this study is the inability to confirm linkage of laterality between the management of the index calcaneus fracture and subsequent clinical outcomes. Although *ICD-10* diagnosis codes used for displaced intra-articular calcaneus fractures (*ICD-10*: S92.061, S92.062, S92.063) specify laterality, the selected outcome codes do not. In particular, posttraumatic osteoarthritis (M19.17) and subtalar fusion procedure codes (28725) are not linked to the index fracture laterality. This likely inflates complication rates in both the operative and Nonoperative cohorts, and future large-scale database studies should use platforms that allow laterality linkage Appendix 1.

Finally, the exclusion of patients with concomitant lower limb fractures may limit generalizability, as many calcaneus fractures occur in the setting of polytrauma.^30^

## Conclusion

Nonoperative management of intra-articular calcaneus fractures was associated with lower rates of both short-term adverse events and long-term sequelae in our cohort. However, these findings are associative and should be considered cautiously because of the inability to control for certain administrative limitations like lack of injury severity and treatment details, which may confound outcomes. We suspect that patients with more minor injuries were biased toward conservative management, and we feel these findings can contribute to patient and surgeon expectations for both strategies when appropriately indicated. Although operative fixation remains a widely used approach, these findings highlight the importance of carefully weighing risks and benefits when deciding on an optimal treatment strategy, and future prospective studies with detailed fracture classification are warranted to better inform clinical decision making.

## Supplemental Material

sj-pdf-1-fao-10.1177_24730114261450622 – Supplemental material for Nonoperative Treatment of Intra-articular Calcaneus Fractures Is Associated With Lower Rates of Certain Long-term Adverse Events Compared to Operative Management: A Retrospective Database Analysis Supplemental material, sj-pdf-1-fao-10.1177_24730114261450622 for Nonoperative Treatment of Intra-articular Calcaneus Fractures Is Associated With Lower Rates of Certain Long-term Adverse Events Compared to Operative Management: A Retrospective Database Analysis by Ava M. McKane, N. Reid Kiritsis, Karl V. Mohr, Omkar S. Anaspure, Conor N. O’Neill and Albert T. Anastasio in Foot & Ankle Orthopaedics

## Appendix

**Appendix 1.** Queried Complications and Their Respective *ICD-10* Diagnosis Codes.

	*ICD-10* / *CPT* Code(s)
Any adverse event	T81.3, I26, I21, I63, I82.40, L76.22, T81.4, A41.9, J18; *ICD-10-PCS*: 30233N1
Dehiscence	T81.3
Transfusion	*ICD-10-PCS*: 30233N1
Acute myocardial infarction (MI)	I21
Cerebral infarct	I63
Pulmonary embolism (PE)	I26
Deep vein thrombosis (DVT)	I82.40
Surgical site infection (SSI)	T81.4
Emergency department visit	*CPT*: 99281, 99282, 99283, 99284, 99285
Hematoma	L76.22
Sepsis	A41.9
Venous thromboembolism (DVT or PE)	I82.40, I26
Inpatient admission	*CPT*: 99221, 99222, 99223
Infected prosthesis	T84.5, T84.6, T84.7
Prosthesis mechanical complication	T84.0, T84.1, T84.2
Delayed healing / nonunion / malunion	S92.002G, S92.001G, S92.009G, S92.062G, S92.061G, S92.063G, S92.001K, S92.002K, S92.009K, S92.061K, S92.062K, S92.063K, S99.002K, S99.001K, S99.009K, S99.091K, S92.001P, S92.002P, S92.009P, S92.061P, S92.062P, S92.063P, S99.092P, S99.002P, S99.001P
Posttraumatic osteoarthritis (foot/ankle)	M19.17
Conversion to subtalar fusion	28725

Abbreviations: *CPT*, *Current Procedural Terminology; ICD-10*, *International Classification of Diseases, Tenth Revision, Procedure Coding System*.
